# Cultural factors weaken but do not reverse left-to-right spatial biases in numerosity processing: Data from Arabic and English monoliterates and Arabic-English biliterates

**DOI:** 10.1371/journal.pone.0261146

**Published:** 2021-12-16

**Authors:** Dominique Lopiccolo, Charles B. Chang

**Affiliations:** Department of Linguistics, Boston University, Boston, Massachusetts, United States of America; French National Center for Scientific Research (CNRS) & University of Lyon, FRANCE

## Abstract

Directional response biases due to a conceptual link between space and number, such as a left-to-right hand bias for increasing numerical magnitude, are known as the SNARC (Spatial-Numerical Association of Response Codes) effect. We investigated how the SNARC effect for numerosities would be influenced by reading-writing direction, task instructions, and ambient visual environment in four literate populations exemplifying opposite reading-writing cultures—namely, Arabic (right-to-left script) and English (left-to-right script). Monoliterates and biliterates in Jordan and the U.S. completed a speeded numerosity comparison task to assess the directionality and magnitude of a SNARC effect in their numerosity processing. Monoliterates’ results replicated previously documented effects of reading-writing direction and task instructions: the SNARC effect found in left-to-right readers was weakened in right-to-left readers, and the left-to-right group exhibited a task-dependency effect (SNARC effect in the *smaller* condition, reverse SNARC effect in the *larger* condition). Biliterates’ results did not show a clear effect of environment; instead, both biliterate groups resembled English monoliterates in showing a left-to-right, task-dependent SNARC effect, albeit weaker than English monoliterates’. The absence of significant biases in all Arabic-reading groups (biliterates and Arabic monoliterates) points to a potential conflict between distinct spatial-numerical mapping codes. This view is explained in terms of the proposed Multiple Competing Codes Theory (MCCT), which posits three distinct spatial-numerical mapping codes (innate, cardinal, ordinal) during numerical processing—each involved at varying levels depending on individual and task factors.

## Introduction

### Mapping codes for numerical representation and access

Humans develop the ability to quantify object sets (henceforth, “numerosities”) early in life, and the early conceptualization of numerosities is thought to form the basis for knowledge of symbolic numbers (e.g., *7*, *seven*) [1–4] (cf. [5] for an alternative view). Given the apparent connection between symbolic and nonsymbolic numerical concepts [6, 7], as well as the prevalence of numeracy across the world’s cultures [8], the processing of symbolic numbers in turn holds insights for our understanding of numerosity processing. A seminal finding of work on symbolic number processing is the so-called SNARC (Spatial-Numerical Association of Response Codes) effect, which refers to a disparity in which small numerical magnitudes are responded to faster with the left hand and large numerical magnitudes with the right [9], consistent with a spatial representation of symbolic digits (i.e., a mental number line going from left to right). Since [9], the SNARC effect has been observed across a wide range of subject populations (including animals [10–13], preverbal infants [14, 15], illiterate individuals [16–18], and biliterate individuals [19, 20]), task paradigms (including parity judgment [9, 21, 22], magnitude comparison [23–26], ordering [27, 28], calculation [29, 30], and line bisection [31–33]), and stimuli (including digits [34–36], number words [9, 37], numerosities [38, 39], and non-numerical magnitudes [40, 41]), albeit with some important differences in the magnitude and directionality of the effect.

The proliferation of research into the SNARC effect has led to several questions regarding the factors involved in numerical (symbolic and nonsymbolic) processing, such as mapping “codes”. The SNARC effect was first explained in terms of numerical representations organized by a cardinal code in long-term memory [9, 42, 43] and later in terms of numerical representations organized by an ordinal code in working memory [44]. Note that, according to [45], “[c]ardinality refers to the total number of items in a set and is tied to concept of numerical magnitude, whereas ordinality refers to numerical sequencing and is relative in nature” (p. 185); therefore, “cardinal code(s)” and “ordinal code(s)”, respectively, are the spatial-numerical associations that form under these distinct order mappings. By extension, cardinal coding is the ordering of numerical stimuli along a small-to-large parameter in conceptual space (i.e., the mental number line), whereas ordinal coding is ordering along a task-based parameter (as [46] shows, these two codes may be at work simultaneously, but their relative strengths are determined by contextual factors). What led [44] to the ordinal code account were findings from a numerical parity judgment task in which participants memorized a sequence of digits, then judged parity for only those digits that were present in the given sequence. The results of this task showed no significant interaction between numerical magnitude and hand/side of response (i.e., no SNARC effect); instead, numbers at the beginning of the sequence were judged more quickly with the left hand and numbers at the end of the sequence with the right, thus reflecting an influence of ordinal information on response biases.

Ordinal coding has also been observed for numerosities. For instance, Patro and Shaki [26] conducted a pairwise comparison task in which participants had to select which of two numerosities was *smaller* (containing fewer items in the set) or *larger* (containing more items in the set). In the *smaller* condition, Polish participants exhibited a typical left-to-right (LR) SNARC effect; however, in the *larger* condition, they tended to show the opposite, right-to-left (RL) response bias, although this tendency did not reach statistical significance. This task-dependency effect showed that numerosities could be mapped according to either increasing or decreasing magnitude, depending on the task instructions.

Although ordinal coding plays a role in symbolic and nonsymbolic numerical processing, it is not always mutually exclusive with cardinal coding. For instance, [46] found different response biases in a magnitude comparison task depending on whether or not participants had to maintain in working memory a five-digit sequence (an ordinal effect if yes, but a cardinal effect—that is, the typical SNARC effect—if no) and, crucially, both biases simultaneously when the two conditions were intermixed. Additionally, [41], using a similar pairwise comparison task as [26], found no task-dependency effect for digits but a strong task-dependency effect for non-numerical magnitudes. Taken together, the results of [26, 41] point to an interaction between cardinal and ordinal codes for numerosities, in that alignment of the two codes (in a “choose *smaller*” task) is linked to a robust directional response bias whereas conflict between the two codes (in a “choose *larger*” task) is linked to a weak bias. In the case of digits, however, conflict between codes still leads to a clear SNARC effect [41], owing to symbolic numbers’ more direct (and, thus, faster) access to the cardinal code, which prevents ordinal coding from taking effect [47–50]. These findings paint a picture in which numerical processing draws upon both the cardinal code and the ordinal code, with task context and stimulus type determining their relative weights.

As studies on the SNARC effect began to include RL script users [17, 18, 41, 51, 52], researchers observed a different spatial-numerical mapping bias. More specifically, RL script users tended to respond more quickly to small numerical magnitudes on the right and large ones on the left—a “reverse” SNARC effect. This “culture effect”, as it is often described [45, 53], seems to influence both cardinal and ordinal codes. For example, a culture effect was observed in Arabs (RL script users) for both symbolic digits and animal pair sizes [41]. Notably, when Arabs switched from a “choose smaller” task to a “choose larger” task, their ordinal code reflected a RL bias, such that they responded more quickly to large animal pair sizes on the right and small ones on the left. Thus, the Arabs’ cardinal code for symbolic digits was effectively RL as evidenced by their reverse SNARC effect, and their ordinal code for animal pair sizes was also RL as evidenced by their task-dependent reverse SNARC effect (i.e., a RL SNARC effect during the “choose smaller” task but a LR SNARC effect during the “choose larger” task).

In addition to culturally-mediated cardinal and ordinal codes, a third spatial-numerical mapping code has been observed in numerical processing: the innate (biological) code. The innate code refers to a spatial bias, prior to the development of numeracy and regardless of culture, to orient increasing numerical magnitudes along a LR axis (cf. [54] for an alternative interpretation of this LR bias). For example, a LR bias was observed in human newborns during a habituation paradigm with bilaterally presented numerosities [15]. Similar LR spatial-numerical associations have been observed in other infant studies [14, 55, 56] as well as animal studies [11, 12, 57], consistent with an innate LR code for increasing numerical magnitude. Further, a growing number of studies that have found LR or null SNARC effects in adult RL script users points toward an innate LR code that may be as strong as, if not stronger than, RL cardinal and ordinal codes. For example, Arabic (RL) and French (LR) readers showed similar LR biases in a temporal order judgment task following a mental arithmetic task [36], while Hebrew (RL) readers also showed a significant LR SNARC effect in a numerical parity judgment task [58].

### Environment, script direction, and bidirectional biliteracy

Although there is now a considerable body of research examining the culture effect on numerical processing [45, 59], this research has often conflated effects of ambient visual environment and script direction, rather than considering these effects separately. The motivation for the latter comes from [9], in which Iranians (RL readers) living in France (a LR culture) showed, in numerical parity judgment, a lack of SNARC effects at the group level, but at the individual level a positive correlation between length of residence in France and magnitude of the SNARC effect. These results supported the conclusion that ambient visual environment plays a role in spatial-numerical associations that is distinct from that of script direction.

This conclusion, however, is not as straightforward as it might appear, due to the possibility of bidirectional biliteracy (i.e., knowledge of two opposing script directions, in this case RL and LR) that was not accounted for. In particular, because the Iranian participants in [9] were highly educated (from medical schools at Parisian universities), they were likely to be proficient in, and even frequent users of, both RL and LR languages, including numerical systems. If they were indeed Persian-French bilinguals (more specifically, biliterates), their bidirectional biliteracy could have prevented strong SNARC effects from arising in either direction; unfortunately, however, their language background was not specified, so one cannot know for sure. More generally, the under-specification of bidirectional biliteracy provides a potential explanation for the lack of (reverse) SNARC effects previously observed in RL populations [17, 26, 51].

Crucially, those studies that tested bidirectional biliterates as such were also carried out in bidirectional visual environments such as Israel [19, 20] and Lebanon [17], making it ambiguous whether the biliterates’ behavior should be attributed to biliteracy, the environment, or both. It also remains unclear whether the LR biases found in some RL script users resulted from a dominant innate LR code overriding RL cardinal and ordinal codes or from the combination of the innate LR code, LR script knowledge (that went unmentioned), and/or LR scanning biases due to the ambient visual environment. In the current study, we looked specifically at bidirectional biliterates living in unidirectional visual environments to help tease apart the effects of environment and (bi)literacy.

### Research questions and hypotheses

With the goal of shedding light on the various spatial-numerical mapping codes involved in numerosity processing, the current study replicated [26], to our knowledge the most recent study of numerosity processing including opposing script directions, with some modifications meant to allow certain response biases to show up more robustly. Our first question (Q1), focused on the culturally-mediated cardinal code, was whether LR and RL monoliterates would show opposite directional response biases for numerosities, as was hypothesized but not clearly observed in [26] for Polish and Hebrew monoliterates. Instead of Hebrew monoliterates, we examined Arabic monoliterates because Arabic, which uses the RL Eastern Arabic numeral system (e.g., 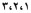) instead of the LR Western Arabic numeral system [8], involves a more consistently RL script direction across words and numbers. This design thus provided a sharper contrast between RL script users and LR script users (in our case, English readers), giving the cardinal code a greater chance to be observed. (To clarify the Western vs. Eastern distinction referenced above, note that the symbolic numerical system of so-called “Arabic numerals”—e.g., 1, 2, 3—is a system that was actually created in India, adopted by Arabs, and taken up by Italian laymen through interactions with Eastern merchants. This system eventually worked its way into higher social classes in Western Europe; therefore, it came to be known as the Western Arabic number system. The Eastern Arabic number system also originated in India and was adopted by Arabs; however, this system was taken up by Islamic leaders in the Middle East and was shaped by the dominant language of Arabic. Since Arabic is written and read in the RL direction, Eastern Arabic numerals are also. The one directional inconsistency in the Eastern Arabic number system is in the orientation of place values: place value decreases in the LR direction just as in the Western Arabic number system. See [8] for a more in-depth explanation of the two numerical systems).

Our second question (Q2), focused on the culturally-mediated ordinal code, was whether participants would show a task-dependency effect in their numerosity processing. To address Q2, we followed [26] in using the pairwise numerosity comparison task with different instructional conditions to test whether changing the task instructions would lead to a change in the occurrence and/or magnitude of an observed SNARC effect. Unlike [26], we used a within-participants design (resulting in data for each condition coming from the same participants) so as to increase the chance of observing a task-dependency effect, which would demonstrate the strength of ordinal coding over cardinal coding in numerosity processing.

Recall that the above-mentioned culture effect may be influenced by script knowledge and/or the ambient visual environment. To help tease these two factors apart, our final question (Q3) asked whether bidirectional biliterates (intentionally balanced in LR/RL script knowledge) would show response biases more similar to monoliterate counterparts living in the same environment (which would imply the predominance of environment) or to other biliterates living in the complementary environment (which would imply the predominance of script direction). To address Q3, we compared two groups of Arabic-English biliterates: those living in Jordan (AEBJOs) and those living in the U.S. (AEBUSs).

Our hypotheses were based in part on a theory we call Multiple Competing Codes Theory (MCCT), which posits that the innate code, the cardinal code, and the ordinal (task-based) code are all at work during numerosity processing, with stimulus type and participant demographics (e.g., culture) determining the relative weighting of these codes. Since [26] compared groups whose script directions were not fully opposing (i.e., Israelis read words RL but numbers LR, a confound acknowledged in recent literature [26, 60, 61]), this comparison might have masked culture effects. Consequently, in regard to Q1, we hypothesized that the comparison of Arabic monoliterates (AMs) and English monoliterates (EMs) would show the influence of a culture effect more clearly, meaning that these groups would indeed show opposite response biases (H1). Furthermore, since numerosity processing has previously been shown to be influenced by both cardinal and ordinal codes, we expected to find a task-dependency effect in the Results of the numerosity comparison task, with stronger directional biases in the condition where the cardinal and ordinal codes are aligned (i.e., where the directional order designated by the task instructions matches the directional order designated by numerosity size) compared to the condition where they are not [46]. Thus, we hypothesized that, as in [26], monoliterates would show a strong directional bias when selecting “smaller” but a weak bias when selecting “larger” (H2).

Finally, since this is, to our knowledge, the first study to explicitly tease apart environment and script knowledge effects in bidirectional biliterates, we considered two potential outcomes in regard to Q3. On the one hand, if ambient visual environment were to play the dominant role in shaping spatial-numerical associations, bidirectional biliterates would show biases closest to those of the monoliterate counterpart living in the same environment. On the other hand, if script direction(s) were to play the dominant role, they would show biases closest to those of other biliterates (regardless of environment). While [9] found a significant environment effect on Farsi readers’ SNARC biases, a more recent study [36] failed to observe this effect on Arabic readers’ biases. Notably, however, the Farsi readers in [9] were likely much more fluent in a LR script than the Arabic readers in [36], pointing to the possibility that an environment effect may not be detectable until a relatively high level of LR script knowledge is attained. Based on these findings and our recruitment of highly proficient, balanced biliterates, we hypothesized that our Arabic-English biliterates would show biases like those seen in [9]. That is, Arabic-English biliterates were hypothesized to show a significant environment effect, meaning that AEBJOs’ biases would pattern more closely with AMs’ and AEBUSs’ biases with EMs’ (H3).

## Materials and methods

The study was approved by the Boston University Charles River Campus Institutional Review Board under protocol #4954E. To determine the number of participants to recruit for the study, we carried out a power analysis anticipating multiple linear regression models with up to 23 coefficients apart from the intercept (accounting for group, condition, and pair size predictors, along with all possible interaction coefficients) and assuming 80% power, an alpha level of.05, and a model *R*^2^ of.165 (based on exploratory modeling of pilot data). Using pwr.f2.test() in the pwr package [62] in R [63], we determined the target number of observations (condition completions) in a between-participants design (i.e., where each participant completes only one condition) to be approximately 153. Therefore, since we used a within-participants design (i.e., where each participant completes both conditions), we recruited participants until we reached a final sample of at least 20 participants in each group (20 participants x 2 conditions x 4 groups = 160 condition completions). Informed consent was obtained orally from all participants prior to their entrance into the study to avoid exposing them to written language prior to the pairwise comparison task; the use of oral consent was approved by the Institutional Review Board, and oral consent was documented by the first author on a participant log.

### Participants

A total of 212 participants completed the study, with 62 excluded from analysis due to failure to follow instructions, obvious misunderstanding of the instructions (i.e., accuracy < 50% in either condition), illiteracy, a residential or educational history inconsistent with the targets for their group, or equipment failure. Thus, the final results included 150 participants, who all reported normal or corrected-to-normal vision in a detailed background questionnaire (the background questionnaire and the MATLAB script described in the next section are publicly available via the Open Science Framework at https://osf.io/yvjw3/). On the basis of their questionnaire responses pertaining to language experience and residential history, participants were each assigned to one of four groups: Arabic monoliterate (AM), English monoliterate (EM), Arabic-English biliterate in Jordan (AEBJO), and Arabic-English biliterate in the U.S. (AEBUS). The AM group (*N* = 21; 14f, 7m; *M*_*age*_ = 37.2 yr, *SD* = 11.4) was recruited from the cities of Mafraq and Zarqa in Jordan. All AMs reported little to no formal training in a LR script, and on average they rated their reading (r) and writing (w) abilities in a LR script as “poor” to “fair” (*M*_*r*_ = 1.6/5, *M*_*w*_ = 1.6/5). The EM group (*N* = 48; 27f, 21m; *M*_*age*_ = 31.5 yr, *SD* = 11.9) was recruited from the cities of Boston (Massachusetts) and Muncie (Indiana) in the U.S. and reported no exposure to or training in a RL script.

To control for environment in the biliterate-monoliterate comparisons, biliterates were recruited from the same regions as their monoliterate peers (AEBJOs from Mafraq, Zarqa, and Amman in Jordan, AEBUSs from Boston and Muncie in the U.S.). With one exception (who started acquiring Arabic at age 6), all biliterate participants identified Arabic as a first language (L1) and had a length of continuous residence of at least one year preceding the time of testing in the target environment: Jordan or another Arabic-speaking country for the AEBJO group (*N* = 31; 14f, 17m; *M*_*age*_ = 31.0 yr, *SD* = 9.5) and the U.S. for the AEBUS group (*N* = 50; 20f, 30m; *M*_*age*_ = 35.8 yr, *SD* = 15.2). AEBJOs and AEBUSs all reported daily usage of both Arabic and English, skewed toward Arabic for most AEBJOs and toward English for most AEBUSs. On average, AEBJOs rated their reading and writing abilities as “native-like” in Arabic (*M*_*r*_ = 4.9/5, *M*_*w*_ = 4.9/5) and “good” to “very good” in English (*M*_*r*_ = 3.9/5, *M*_*w*_ = 3.6/5); similarly, AEBUSs rated their reading and writing abilities as “very good” to “native-like” in Arabic (*M*_*r*_ = 4.7/5, *M*_*w*_ = 4.6/5) and “good” to “very good” in English (*M*_*r*_ = 4.2/5, *M*_*w*_ = 3.9/5). None of the between-group differences in self-ratings was significant [Wilcoxon |*W*|s < 877, n.s.]. As for language of instruction, early schooling was primarily in Arabic for nearly all AEBJOs and several AEBUSs, but in English or both Arabic and English for the majority of AEBUSs.

Because environment is a complex construct, the effect of environmental differences between the two biliterate groups was operationalized for investigation in three different ways. First, we examined the effect of the current environment at the time of study by way of the basic group comparison between AEBJOs and AEBUSs. Second, we examined the effect of cumulative time spent in LR visual environments by calculating for each biliterate participant (on the basis of their residential history) a lifetime length of residence (LOR, in years) in countries where LR scripts are dominant. As expected, these LOR values were significantly longer for AEBUSs (*M* = 14.2) than for AEBJOs (*M* = 1.1) [Wilcoxon *W* = 1476.5, *p* < .001]. Finally, we examined the effect of preferred language for counting (CountLang), a relevant variable that was highly correlated with current environment: as expected, CountLang for AEBJOs was Arabic for most (61%), English for some (32%), and both Arabic and English for a few (7%), whereas for AEBUSs it was English for most (65%), Arabic for some (25%), and both Arabic and English for a few (10%). As with LOR, the disparity in CountLang between AEBJOs and AEBUSs was statistically significant [Wilcoxon *W* = 1046.0, *p* < .001].

### Stimuli

The focal task in the study involved comparison of numerosities (i.e., arrays of dots), which were created using [64]’s MATLAB script for generating numerosities. Written specifically for experimental paradigms such as pairwise comparison, this script randomizes the values of continuous variables such as dot size in such a way as to limit participants’ reliance on irrelevant features to make quantity judgments. For example, a continuous variable might sometimes be made to be directly related to the size of the generated numerosity (e.g., larger dot size for larger numerosities) and sometimes inversely related (e.g., smaller dot size for larger numerosities), thereby reducing the likelihood of this variable having an extraneous effect on quantity judgments.

**Fig 1:**
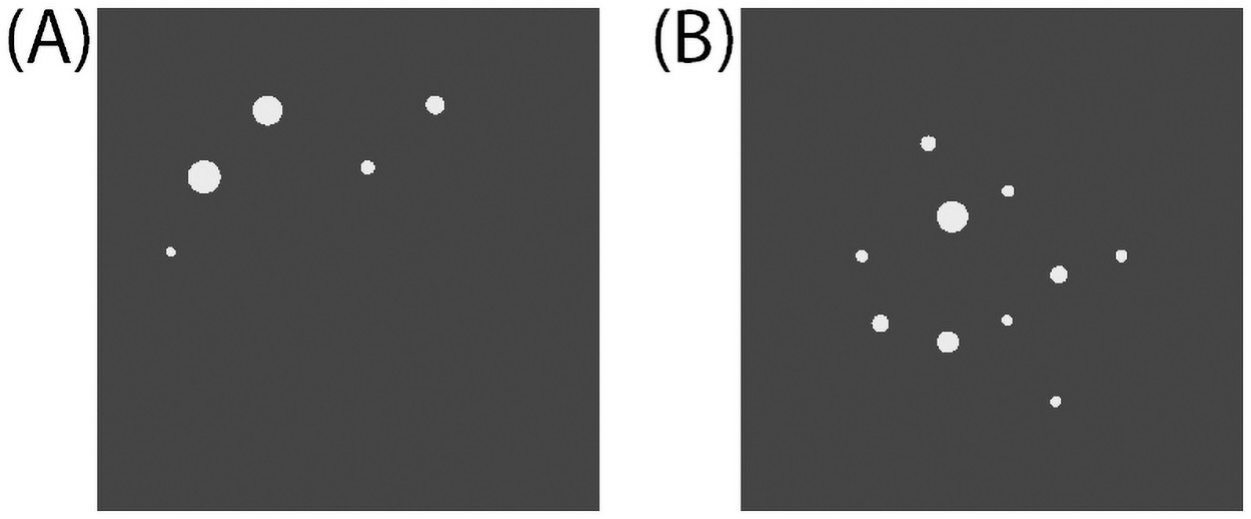
Sample stimuli in the pairwise numerosity comparison task. A: Example array for numerosity 5. B: Example array for numerosity 10. The stimuli pictured exemplify a representation of the “large” pair 5–10.

The stimulus set included the nine pairs of numerosities in [26] (see p. 130 for the rationale behind these pairs): 2–4, 2–3, 3–4, 3–6, 4–6, 4–8, 5–10, 6–8, and 6–9. Following [26]’s design, these pairs were classified by size as “small” (quantity 2–4), “large” (quantity 5–10), or intermediate “cross-range” (mean quantity of 3.0, 7.3, and 5.2, respectively). During the experiment, each pair was displayed with four unique representations (i.e., physically different pairs of dot arrays), one in the practice trials and three in the test trials. Fig 1 shows an example pair of numerosities representing the large pair 5–10.

Note that small and cross-range, but not large, pairs contained at least one numerosity within the “subitizing” range, which typically includes numerosities 1–4 and involves rapid processing mechanisms that are unique to this range [65, 66]. This disparity between pair sizes could pose a problem for the interpretation of differences observed in the Results. Nevertheless, because effects of the subitizing range are not clear-cut [67–69] and the purpose of our study was to build directly upon [26]’s findings, we decided to continue using the same numerosity pairs as [26] (rather than, e.g., using all “large” pairs), with careful consideration of the different processing mechanisms that may be involved for this set of pairs. We return to this point in the Discussion.

### Procedure

Participants were tested individually in the country where they were recruited (Jordan for AMs and AEBJOs, and the U.S. for EMs and AEBUSs) and completed all parts of the study in a quiet room in one session. The study consisted of two parts, completed in the following order: (1) a speeded (i.e., respond as quickly as possible) bilateral pairwise comparison task with numerosities, and (2) a detailed background questionnaire, which included items about residential history, language proficiencies, and handedness (based on the Edinburgh Handedness Survey [70]). The questionnaire was administered in Arabic to AMs and AEBJOs and in English to EMs and AEBUSs.

Each participant completed both conditions of the pairwise comparison task (with condition order counterbalanced across participants), the *smaller* condition (i.e., select the smaller numerosity of the two presented) and the *larger* condition. Both conditions consisted of an initial 18-trial practice block with audio-only corrective feedback and three 18-trial test blocks without corrective feedback; optional breaks were included at the end of each block. Each unique pair of dot arrays was presented twice with different bilateral orientations (i.e., smaller numerosity on the left in one trial and on the right in the other trial). This design resulted in 72 total practice and test trials (9 numerosity pairs x 4 unique representations x 2 orientations) in each condition.

Because participant exclusions did not occur evenly across the two possible condition orders (resulting in uneven counterbalancing across condition orders in some groups), we checked to see whether condition order had a significant effect on the dependent variables, response time (RT) and accuracy. A likelihood ratio test of two linear mixed-effects models (with random intercepts by Participant and Pair) with and without a term for Condition Order showed no effect of Condition Order on RT [*χ*^2^(1) = 0.690, n.s.]. Similarly, a likelihood ratio test of corresponding logistic mixed-effects models showed no effect of Condition Order on the log odds of accuracy [*χ*^2^(1) = 0.012, n.s.]. Thus, disparities in counterbalancing are unlikely to account for the between-group differences observed in the pairwise comparison task.

The pairwise comparison task was completed on a MacBook Pro laptop running OpenSesame 3.2.6 [71], using a Cedrus 7-button response pad (RB-740) and studio-quality binaural headphones. To make the instructions an extension of the ambient language environment, initial audio-only instructions were played in Modern Standard Arabic for AMs and AEBJOs and in Mainstream U.S. English for EMs and AEBUSs. Participants placed the dominant finger (in most cases, this was their index finger) of each hand on the left and right target response buttons, respectively, and began the task by pressing either button. Their task was to—as quickly and accurately as possible—press the left button if the numerosity on the left side of the screen was the target and the right button if the one on the right side of the screen was the target.

## Results

The results reported below concern the central analysis of differences in response times (RTs) by group, pair size, and condition, as well as additional models of RT differences that operationalized environment in terms of either lifetime length of residence (LOR) in LR-script environments (cf. [9]) or preferred language for counting (CountLang). For reasons of space, we do not report here error rate analyses as in [26]; note, however, that we did carry out error rate analyses, which generally corroborated the results of Models 1A–1D described below. These latter analyses, along with the full dataset and a summary table of raw RTs, can be viewed on the Open Science Framework at https://osf.io/7rgf8/.

### Effects of group, pair size, and condition

Our first set of analyses focused on RT differences from the test blocks and excluded RTs that were for incorrect responses, less than 150 ms (indicating a response that was unlikely to have been to the stimuli), or outliers more than 3 *SD* from individual participant means (= 3.2% of the data). In a linear mixed-effects model (with a treatment-coded Size predictor and random intercepts by Participant and Target Side), built using lmer() in the lmerTest package [72] in R, raw RTs showed a “size effect” [73]: RTs were significantly longer on large pairs [*β* = 61.865, *t* = 16.296, *p* < .001] and shorter on cross-range pairs [*β* = −11.127, *t* = −2.961, *p* = .003] compared to small pairs. In addition, mean RTs showed a negative correlation with error rates [*r*(147) = −.161, *t* = −1.977, *p* = .050], suggesting a slight trade-off between speed and accuracy. We calculated the dependent variable of RT difference by pair, subtracting a participant’s mean left-hand RT for a given pair (e.g., 2–3) from their mean right-hand RT for that pair (see [26] for rationale). Thus, a positive RT difference indicates a left-hand bias while a negative RT difference indicates a right-hand bias.

**Fig 2:**
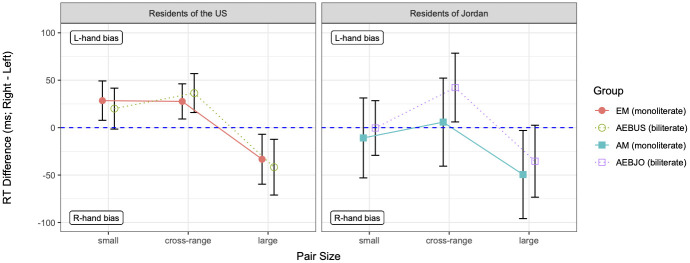
Mean RT differences (in milliseconds) in the *smaller* condition, by group and pair size. Groups residing in the U.S. are plotted on the left; groups residing in Jordan on the right. Each pair size represents the average over the three pairs for that pair size. The dashed horizontal line marks zero RT difference (i.e., no hand bias); error bars mark 95% confidence intervals.

**Fig 3:**
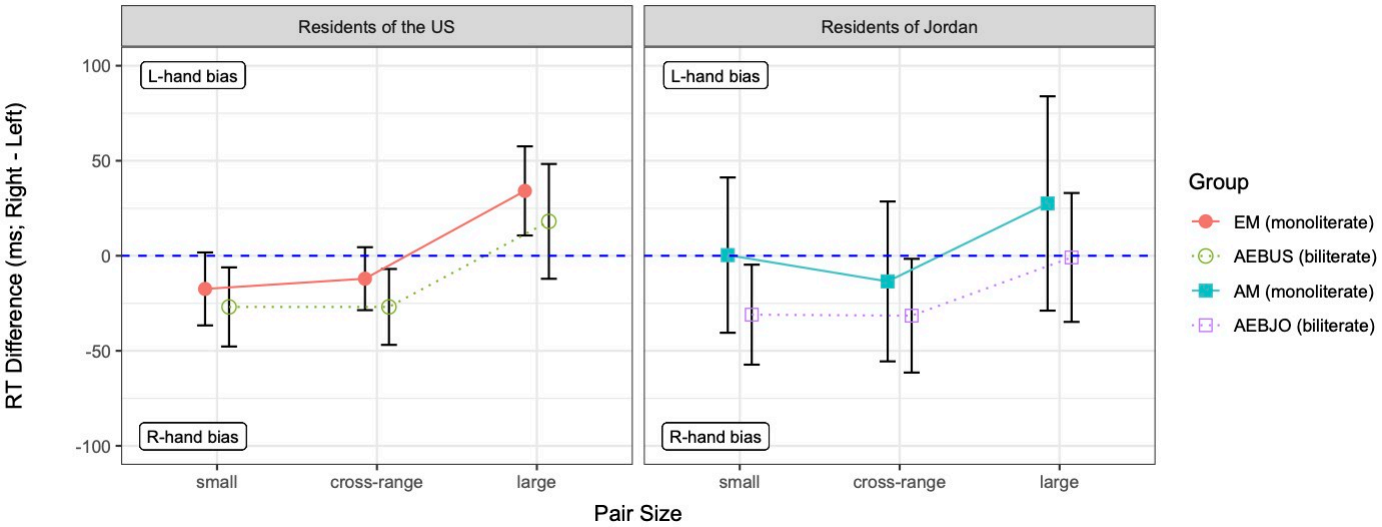
Mean RT differences (in milliseconds) in the *larger* condition, by group and pair size. Groups in the U.S. are plotted on the left; groups in Jordan on the right. Each pair size represents the average over the three pairs for that pair size. The dashed horizontal line marks zero RT difference (i.e., no hand bias); error bars mark 95% confidence intervals.

The RT difference data are graphed in Figs 2 and 3, respectively, for the *smaller* condition and *larger* condition. In the context of graphs of this type, the standard SNARC effect, a LR bias, takes the form of a negative slope with increasing numerosity size (i.e., more negative RT differences for larger numerosities), whereas the reverse SNARC effect, a RL bias, takes the opposite form. As can be seen in Figs 2 and 3, there is indeed a SNARC effect (i.e., an effect of pair size), as well as a change in the SNARC effect across conditions; more broadly, there are also differences and similarities among the groups. To analyze the data statistically, we built a series of linear mixed-effects models, treating each of the four groups as the reference level of a Group predictor.

To address Q1–Q2 concerning SNARC effects and task-dependency effects in LR and RL monoliterates, we first built models focusing on the comparison of the EM and AM groups (Models 1A–1B). These models included treatment-coded fixed effects for Group (reference level = EM in Model 1A, AM in Model 1B), (Pair) Size (reference level = small), and Condition (reference level = *smaller*), and random intercepts by Participant and Pair (for rationale, see [74]). The models also included terms for all possible interactions among the fixed-effect predictors. An analysis of variance (ANOVA) on Model 1A (using Anova() in the car package [75]) revealed no main effect of Group, Size, or Condition and no Group × Size or Group × Size × Condition interaction [*χ*^2^s < 3.022, n.s.]. However, there was a marginal Group × Condition interaction [*χ*^2^(3) = 6.412, *p* = .093], reflecting a tendency for the disparity between conditions in RT differences to vary across groups. There was also a significant Size × Condition interaction [*χ*^2^(2) = 73.667, *p* < .001], reflecting the global change in the nature of the SNARC effect across conditions. The fixed-effect coefficients in Models 1A–1B are summarized in the S1 and S2 Tables.

Starting with the results for the *smaller* condition, as seen in Fig 2, EMs showed a SNARC effect in terms of going from a left-hand bias on small pairs [*β* = 28.483, *t* = 2.142, *p* = .033], which stayed a left-hand bias on cross-range pairs [*β* = −0.825, *t* = −0.046, n.s.], to a stronger right-hand bias (i.e., more negative RT difference) on large pairs [*β* = −61.849, *t* = −3.456, *p* < .001]. On the other hand, AMs showed no hand bias on small pairs [*β* = −10.841, *t* = −0.533, n.s.] and no significant change in bias on cross-range or large pairs [|*β*|s < 38.612, |*t*|s < 1.417, n.s.], resulting in effectively no SNARC effect. Thus, these results partially confirm our first hypothesis, H1: AMs and EMs did in fact have different response biases for numerosities, in that EMs showed a strong LR bias whereas AMs did not. However, AMs did not show the reverse (RL) bias; rather, they showed no significant directional bias.

As for the *larger* condition, the nature of the SNARC effect differed compared to that seen in the *smaller* condition, especially among EMs. As seen in Fig 3, EMs showed a reverse SNARC effect in this condition in terms of going from a stronger right-hand bias on small pairs [*β* = −45.925, *t* = −2.629, *p* = .009], which stayed a right-hand bias on cross-range pairs [*β* = 6.216, *t* = 0.252, n.s.], to a left-hand bias on large pairs [*β* = 113.424, *t* = 4.588, *p* < .001]. However, the magnitude of EMs’ RL bias in the *larger* condition (as reflected in the distance between mean RT differences to large and small pair sizes) was slightly smaller than that of their LR bias in the *smaller* condition (magnitude of RL bias, *larger* condition = 52, cf. magnitude of LR bias, *smaller* condition = 62; see Figs 2 and 3). Meanwhile, AMs also tended to show more of a left-hand bias on large pairs in the *larger* condition compared to the *smaller* condition, but this tendency was not statistically significant [*β* = 65.786, *t* = 1.756, n.s.]. These results therefore partially support our second hypothesis, H2, regarding a task-dependency effect: the SNARC effect for EMs was indeed weakened in the *larger* condition vis-a-vis the *smaller* condition, in that EMs showed a RL bias that was slightly smaller than their LR bias from the *smaller* condition. However, AMs did not show the reverse (here, LR) bias; furthermore, AMs’ bias, which was again weaker than EMs’ and in fact not significant, went in the same direction as EMs’.

To address Q3 concerning SNARC effects in RL-LR biliterates living in different visual environments, we built two additional models focusing on the comparison of the AEBUS and AEBJO groups to the AM and EM groups, as well as the comparison of the AEBUS and AEBJO groups to each other (Models 1C–1D). The structure of each of these models was the same as that of Models 1A–1B except in the coding of the Group factor, which was set to a reference level of AEBUS in Model 1C and AEBJO in Model 1D. The fixed-effect coefficients in Models 1C–1D are summarized in the S3 and S4 Tables.

Starting again with the *smaller* condition, AEBUSs resembled EMs (see Fig 2) in showing a SNARC effect, going from a non-significantly left-skewed bias on small pairs [*β* = 20.106, *t* = 1.472, n.s.], which did not significantly change on cross-range pairs [*β* = 16.351, *t* = 0.890, n.s.], to a stronger right-hand bias on large pairs [*β* = −61.796, *t* = −3.364, *p* = .001]; in fact, the SNARC effect for EMs did not differ significantly [*β* = −0.028, *t* = −0.001, n.s.]. AEBUSs also did not differ significantly from AEBJOs, whose bias on large pairs in this condition was non-significantly less skewed toward the right-hand [*β* = 26.773, *t* = 0.964, n.s.]. AEBJOs still tended toward a stronger right-hand bias on large pairs compared to small pairs like AEBUSs; however, as with AMs, AEBJOs’ bias on large pairs was weaker than AEBUSs’ and not significantly skewed toward the right hand [*β* = −35.023, *t* = −1.540, n.s.]. Thus, although the LR bias tended to be stronger for both AMs and AEBUSs (as reflected in negative interaction coefficients), neither difference with respect to the AEBJOs was significant. In short, in the *smaller* condition, AEBUSs and AEBJOs were numerically more similar to EMs and AMs, respectively, than to each other, but neither tendency to pattern more closely with one monoliterate group over the other biliterate group was statistically significant.

With regard to the *larger* condition, both biliterate groups resembled EMs in showing a RL bias. As evident in the comparison of Figs 2 and 3, AEBUSs showed a stronger right-hand bias on small pairs in the *larger* condition compared to the *smaller* condition [*β* = −47.019, *t* = −2.733, *p* = .006]. Further, AEBUSs patterned like EMs in showing a similar bias on cross-range pairs [*β* = −16.440, *t* = −0.676, n.s.] and a stronger left-hand bias on large pairs [*β* = 106.807, *t* = 4.394, *p* < .001], such that neither of the three-way interaction coefficients with Group = EM was significant (as discussed above, AEBJOs patterned differently in tending to show a weaker directional bias in this condition). Similar to AEBUSs, AEBJOs tended to show a right-hand bias on small pairs, which did not change significantly on cross-range pairs [*β* = −43.186, *t* = −1.400, n.s.], and a stronger left-hand bias on large pairs [*β* = 65.136, *t* = 2.112, *p* = .035]. The RL bias tended to be stronger for both AMs and AEBUSs (as reflected in positive interaction coefficients), to a greater degree for AEBUSs; however, again neither difference with respect to the AEBJOs was significant. Thus, as in the *smaller* condition, in the *larger* condition AEBUSs were more similar to EMs while AEBJOs were more similar to AMs, but only numerically and not statistically.

Additional comparisons of AEBUSs’ and AEBJOs’ response patterns showed more evidence of differences between these groups. Although AEBUSs’ and AEBJOs’ directional biases in the *larger* condition were both RL, AEBUSs’ bias was slightly stronger (distance in mean RT differences to large and small pairs of 45 for AEBUSs vs. 30 for AEBJOs; see Fig 3) and thus the more similar of the two to EMs’. Relative to AEBUSs’ strong LR bias in the *smaller* condition, AEBJOs’ LR bias, like AMs’, was also weaker and not significant [*β* = −35.023, *t* = −1.540, n.s.]. Together, these results provide partial support for our third hypothesis, H3, because only a weak effect of environment was observed overall: as predicted, there were differences between the two biliterate groups, and each biliterate group patterned more like the monoliterate group in the same environment than like the other biliterate group. However, these patterns were subtle, because the number of non-significant hand biases, across all groups and both conditions, limited how much contrast could be observed between the biliterate groups to begin with. We return to this point in the Discussion.

### Effects of residence and counting language

To examine the relationship between biliterates’ lifetime LOR in LR-script environments and their directional response biases, we built an additional mixed-effects model of RT differences in AEBUSs and AEBJOs (Model 2A). Model 2A had the same random-effects structure as Models 1A–1D (random intercepts by Participant and Pair) and nearly the same fixed-effects structure, except that the Group effect was replaced by the LOR effect. LOR values were positively skewed, so for the purposes of this analysis the LOR variable was log-transformed. An ANOVA on Model 2A showed no effect of (log) LOR [*χ*^2^(1) = 0.212, *p* = .645] and no interactions of LOR with Size and/or Condition [*χ*^2^s < 1.387, n.s.]. We followed up this model with a similar model of AEBUSs’ RT differences (Model 2B), which had an identical structure except that the LOR effect represented continuous LOR in the current LR-script environment (i.e., the US) up until the time of study, as in [9]. An ANOVA on Model 2B also showed no effect of (log) LOR [*χ*^2^(1) = 0.874, *p* = .350], no LOR × Size interaction [*χ*^2^(2) = 1.560, *p* = .458], and no LOR × Size × Condition interaction [*χ*^2^(2) = 2.641, *p* = .267], although there was a marginal LOR × Condition interaction [*χ*^2^(1) = 3.091, *p* = .079]. These results thus provided no clear evidence for an effect of environment as operationalized in terms of individuals’ LOR.

To examine the role of linguistic counting preferences linked to environment (CountLang; i.e., participants’ preferred languages for counting), we built another mixed-effects model of all biliterates’ RT differences (Model 3), with the same structure as Models 2A–2B except that the LOR effect was replaced by CountLang (treatment-coded, with reference level ‘English’). In contrast to the models examining LOR effects, Model 3 suggested that CountLang played a significant role in biliterates’ RT differences. An ANOVA on Model 3 showed no main effect of CountLang [*χ*^2^(2) = 0.584, *p* = .747] and no CountLang × Size interaction [*χ*^2^(2) = 4.080, *p* = .395] but a significant CountLang × Condition [*χ*^2^(2) = 8.464, *p* = .015] and marginal CountLang × Size × Condition interaction [*χ*^2^(4) = 9.199, *p* = .056]. The fixed-effect coefficients in Model 3 are summarized in the S5 Table.

**Fig 4:**
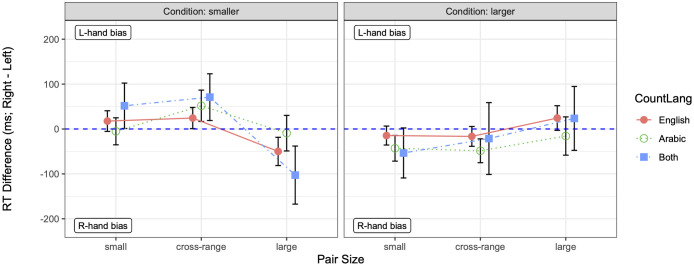
Mean RT differences (in milliseconds), by preferred language(s) for counting (CountLang) and pair size. The *smaller* condition is plotted on the left; the *larger* condition on the right. Each pair size represents the average over the three pairs for that pair size. The dashed horizontal line marks zero RT difference (i.e., no hand bias); error bars mark 95% confidence intervals.

As shown in Fig 4, the nature of the CountLang effect echoed the group differences evident in Models 1A–1D. To be more specific, whereas biliterates who counted in English showed a standard SNARC effect in the *smaller* condition via more negative RT differences on large (but not cross-range) pairs vis-a-vis small pairs [*β* = −67.689, *t* = −3.553, *p* < .001], biliterates who counted in Arabic showed a weaker (namely, null) SNARC effect, as reflected in RT differences that were marginally more positive on cross-range pairs [*β* = 50.204, *t* = 1.735, *p* = .083] and significantly more positive on large pairs [*β* = 63.587, *t* = 2.197, *p* = .028]. On the other hand, biliterates who counted in both English and Arabic tended toward an even stronger SNARC effect, as reflected in RT differences that were marginally more negative on large pairs [*β* = −86.676, *t* = −1.738, *p* = .082]. In the *larger* condition, the SNARC effect for biliterates who counted in English was reversed on large (but not cross-range) pairs [*β* = 106.719, *t* = 4.000, *p* < .001], but this reversal tended to be canceled in biliterates who counted in Arabic [*β* = −75.307, *t* = −1.841, *p* = .066], leading again to a null SNARC effect. Once again, biliterates who counted in both English and Arabic showed the same (reverse) SNARC effect as bilinguals who counted in English, but tended toward an even stronger effect, as reflected in marginally more positive RT differences on large pairs [*β* = 124.654, *t* = 1.768, *p* = .077]. Taken together, these results provided evidence for an indirect effect of environment as mediated by preferred language(s) for counting. Further, a split preference for counting in English or Arabic was, surprisingly, associated with stronger directional response biases than counting in English.

## Discussion

### Synthesis of the findings

The findings of this study partially support our hypotheses (H1–H3) and build upon the results of [26] in three ways. First, looking at more consistently RL script users (AMs) and implementing stricter control of extraneous continuous variables, we obtained results for RT differences that converged with [26]’s: AMs failed to show a significant reverse SNARC effect in either task condition (cf. H1), much like [26]’s Hebrew readers. Second, we found, in our LR monoliterates (EMs), a task-dependency effect that consisted of stronger (reverse) SNARC effects than observed in [26]’s LR monoliterates (Poles): across both task conditions, EMs showed a significant directional bias, which was weaker in the *larger* condition as hypothesized (H2). In short, EMs and AMs showed different, albeit not opposite, response patterns—the former, a task-dependent SNARC effect, and the latter, a null effect. Third, we contributed the first data on numerosity processing by bidirectional biliterates in complementary unidirectional visual environments. The group comparison of U.S.-based biliterates (AEBUSs) and Jordan-based biliterates (AEBJOs) showed only a weak interaction of current environment with biliteracy, with both groups showing broad similarities with EMs suggestive of an innate LR code. In addition, there was no significant effect of length of residence in LR-script environments (although an important caveat here is that the assumption that LOR would have a linear effect may be faulty, since the literature on L1 attrition has suggested that LOR in a second language, or L2, environment may have an effect that is not linear and/or not in the expected direction [76, 77]). On the other hand, preferred language(s) for counting, which differed between AEBUSs and AEBJOs in accordance with their respective environments, did play a significant role. These findings thus point to the conclusion that while environment has, at best, only a weak direct effect on directional biases (cf. H3), it may additionally exert an indirect effect via behavioral outcomes such as linguistic counting preferences that are shaped by environment.

Two additional aspects of our findings are worth noting: RTs were significantly faster, and hand/side biases were generally weaker, on small and cross-range pairs as compared to large pairs. These outcomes are consistent with previous findings showing a link between slower RTs and stronger SNARC effects [78, 79]. The faster RTs observed on small and cross-range pairs in this study are attributable to the rapid enumeration process associated with the subitizing range, which generally refers to numerosities 1–4 and is not culture-specific [16, 80, 81]. Although the mechanism underlying the subitizing range is not entirely understood, and the range itself can vary depending on task demands and individual differences [65, 66, 68], research shows overall faster RTs, higher accuracies, and narrower activation sites for numerosities in the subitizing range [39, 82–84], most likely the result of automatic processing of exact (versus approximate) magnitude.

Crucially, the possibility of subitizing-range processing of small and cross-range pairs means that the current study can be viewed as a strong test of SNARC effects in readers of opposite-direction scripts. That is, given that faster RTs are associated with weaker SNARC effects, the inclusion of pair sizes within the faster-processed subitizing range works against finding SNARC effects. As such, the significant directional biases that were found in this study—in spite of this aspect of the design—can be interpreted as a robust result. Although it was important to follow the same overall design in [26] to facilitate cross-comparison between studies and establish whether SNARC effects occur in bidirectional biliterates to begin with, now that the current findings have laid this groundwork, it would be advantageous to extend this research to more numerosities outside the subitizing range in order to see whether the groups examined in this study show stronger SNARC effects with these larger numerosities [84–86]. Other elements that tend to elicit stronger SNARC effects could be incorporated into the design as well, such as a fixed standard for comparison [87–89], a wider range of numerosities [84], and more repetitions of the same numerosity stimuli [90]. Given that the SNARC effect draws upon relative magnitude processing [34, 89], these design elements tend to evoke stronger response biases because they allow participants to mentally “tag” numerosities as relatively “small” or “large”. Therefore, incorporating these design elements may help reduce noise in spatial-numerical mapping and elicit clear SNARC effects in groups where such effects have generally been found to be weak or absent (i.e., RL readers).

### Accounting for variation in the SNARC effect

Within the framework of Multiple Competing Codes Theory (MCCT), variation in the SNARC effect can be explained in terms of the interaction of potentially conflicting spatial-numerical mapping codes. For example, the task-dependency effect in numerosity processing—in particular, the weaker directional bias shown by some participants in our *larger* condition—is attributable to a conflict in this task condition between the small-to-large ordering of the cardinal code and the large-to-small ordering of the ordinal code. For some individuals, the mismatching cardinal code is not strong enough to mask the ordinal effect (i.e., result in a null bias), consistent with the view that the ordinal code may outweigh the cardinal code in the context of numerosity processing.

Another dimension of variation in the SNARC effect was evident in the absence of significant directional biases in our Jordan-based groups (AMs and AEBJOs), which may be interpreted in various ways. By comparing these groups of RL readers with other RL groups [18–20], we can offer two different, and not mutually exclusive, explanations for their null biases. First, given that LR groups generally show stronger SNARC effects than RL groups in the literature [45, 53], the innate LR code is likely to be contributing to this difference. According to MCCT, the innate code is either strengthened or attenuated as a consequence of formal numeracy and literacy development. Therefore, like [26]’s Hebrew readers, our Arabic readers in Jordan may have shown null biases because of a conflict between RL cultural factors and the innate LR code, which does not occur for LR groups. Second, AMs and AEBJOs may have lacked a consistent response strategy in the pairwise comparison task. Zebian [17] mentions a number of technical safeguards she implemented to ensure her Lebanese participants were familiarized with task requirements before data collection; in retrospect, our study would have benefited from incorporating more of these safeguards. As in [17], AMs and AEBJOs were tested in non-laboratory settings (homes, schools, and other convenient meeting places); nevertheless, misunderstanding of task instructions (in particular, the switch from the *smaller* to the *larger* condition, and vice versa) was most common in these two groups, who represented the majority of the data exclusions due to low accuracies. Consequently, it is possible that these groups approached the task in a fundamentally different way than the U.S.-based groups. Future studies should consider adding a neuroimaging component to inspect the brain activation patterns that occur in these groups during processing, which would help verify whether participants in a RL environment do indeed engage with tasks such as pairwise comparison differently than participants in a LR environment.

Since bidirectional biliterates have not been studied extensively in the field of numerical cognition, there remain several open questions concerning the precise role of dual script directions in introducing variation in the SNARC effect. Recall that our aim in examining two groups of bidirectional biliterates was to tease apart script direction effects from environment effects, in service of elaborating the nature of a more generally described “culture effect”. Given the overall lack of significant biases in these groups, in addition to null effects of length of residence in LR-script environments, we did not find clear evidence of a direct effect of ambient visual environment and are thus not in a position to conclude whether environment or script knowledge plays a more prominent role in the spatial biases observed during numerosity processing. One possibility is that, in the absence of exposure to text during numerosity processing, culture effects weaken, allowing the innate LR code to take over. In future work, it would therefore be worth seeing whether evoking stronger (reverse) SNARC effects by incorporating some of the aforementioned design elements would lead to the same pattern of results.

An additional open question concerns, more generally, the scope of possible interactions among the various spatial-numerical mapping codes available to bidirectional biliterates. Under MCCT, bidirectional biliterates actually have two sets of (potentially conflicting) cardinal and ordinal codes (one for each script direction), meaning that, together with the innate code, they engage with a total of five spatial-numerical mapping codes. Since both biliterate groups in the current study showed biases similar to EMs’ (albeit not statistically significant), it could therefore be the case that their conflicting pairs of ordinal and cardinal codes cancel each other out, but their mutual activation also works to weaken the innate LR code, preventing it from producing robust LR SNARC effects. Further research on bidirectional biliterates using more sensitive task designs for numerosity processing may help elucidate the true strength of the innate LR code as well as the ways in which it may be modulated by other relevant spatial-numerical mapping codes.

On a final note, the indirect effect of environment observed via linguistic counting preferences invites a number of follow-up questions for future work on the SNARC effect in bidirectional biliterates. For one, what are other ways in which environment might indirectly play a role in biliterates’ directional response biases? To take another, what is the mechanism underlying the pattern observed in “bilingual counters” (i.e., biliterates who reported no clear preference of one language over another for counting) whereby their experience counting in Arabic was linked to *stronger* EM-like SNARC effects, in contrast to the weaker SNARC effects observed in AMs? On the one hand, this pattern is consistent with the notion of a robust innate LR code; on the other hand, the presence of an innate LR code does not in itself predict enhanced SNARC effects in bilingual counters. Thus, future work examining bidirectional biliterates as bilinguals, which takes advantage of experimental paradigms manipulating relevant factors such as “language mode” [91], may offer insight into how the flexibility and context-sensitivity of bilingual language systems may lead to numerosity processing outcomes distinct from those of monolinguals/monoliterates.

## Conclusion

The current findings connect with the broader literature on numerical cognition in three ways. First, new data from monoliterates and bidirectional biliterates suggest that nonsymbolic numerical magnitudes (numerosities) maintain shared and separate processing mechanisms (“spatial-numerical mapping codes” in MCCT) vis-a-vis symbolic numerical magnitudes (digits). Digits and numerosities maintain a shared abstract representational system for numerical magnitude, reflected in the influence of the cardinal code for both; however, their pathways to accessing this representational system differ, as evident in disparate ordinal effects (e.g., the task-dependency effect in the current study vs. no task-dependency effect in [41]). Second, the finding of no robust (reverse) SNARC effects in Arabic monoliterates converges with previous findings of weak SNARC effects in RL groups, as well as with findings of early LR biases in infant and animal studies [11, 14], in supporting the existence of an innate LR code. Third, the absence of a clear direct effect of environment in bidirectional biliterates further suggests that the innate LR code is robust, consistent with recent findings on LR biases in RL script users [36, 60, 92]. In short, our findings point to a complex configuration of factors (and variable weights) influencing directional response biases during numerosity processing, one that resembles in broad strokes the configuration of factors involved during symbolic numerical processing.

Given that this was, to our knowledge, the first study of numerosity processing to examine bidirectional biliterates in different locations along with their monoliterate counterparts, there are several directions for further research. For one, more cross-cultural neuroimaging research is needed to see whether the observed behavioral differences between groups (e.g., in task-dependency effects) are reflected in distinct brain activation patterns. Moreover, whereas we have assumed a unidirectional kind of influence from engaging with a script, the reality is that a reader’s eyes typically jump around during reading, raising the question of what type of effect is exerted by such excursions and inconsistencies in visual scanning (cf. [93]); homing in on the most proximal scanning experiences associated with reading would thus help round out the picture of script direction effects. In closing, much remains to be discovered about the role of (bi)literacy in numerical processing, making this a fertile area for future work in numerical cognition.

## Supporting information

S1 Table Fixed effects in Model 1A (intercept represents Group = EM, Size = small, Condition = *smaller*). (PDF)

S2 Table Fixed effects in Model 1B (intercept represents Group = AM, Size = small, Condition = *smaller*). (PDF)

S3 Table Fixed effects in Model 1C (intercept represents Group = AEBUS, Size = small, Condition = *smaller*). (PDF)

S4 Table Fixed effects in Model 1D (intercept represents Group = AEBJO, Size = small, Condition = *smaller*). (PDF)

S5 Table Fixed effects in Model 3 (intercept represents CountLang = English, Size = small, Condition = *smaller*). (PDF)

## References

[1] Gallistel CR, Gelman R. Preverbal and Verbal Counting and Computation. Cognition. 1992;44(1–2):43–74. doi: 10.1016/0010-0277(92)90050-R 1511586

[2] Carey S. Where Our Number Concepts Come From. The Journal of Philosophy. 2009;106(4):220–254. 23136450 10.5840/jphil2009106418 PMC3489488

[3] Dehaene S. The Number Sense: How the Mind Creates Mathematics. 2nd ed. New York, NY: Oxford University Press; 2011.

[4] Vallortigara G. Comparative Cognition of Number and Space: The Case of Geometry and of the Mental Number Line. Philosophical Transactions of the Royal Society B: Biological Sciences. 2018;373(1740):20170120. doi: 10.1098/rstb.2017.0120 PMC5784052 29292353

[5] Marinova M, Sasanguie D, Reynvoet B. Numerals Do Not Need Numerosities: Robust Evidence for Distinct Numerical Representations for Symbolic and Non-Symbolic Numbers. Psychological Research. 2021;85(2):764–776. doi: 10.1007/s00426-019-01286-z 31953564

[6] Szkudlarek E, Brannon EM. Does the Approximate Number System Serve as a Foundation for Symbolic Mathematics? Language Learning and Development. 2017;13(2):171–190. doi: 10.1080/15475441.2016.1263573 28344520 PMC5362122

[7] Wilkey ED, Ansari D. Challenging the Neurobiological Link between Number Sense and Symbolic Numerical Abilities. Annals of the New York Academy of Sciences. 2020;1464(1):76–98. doi: 10.1111/nyas.14225 31549430

[8] Menninger K. Number Words and Number Symbols: A Cultural History of Numbers. New York, NY: Dover Publications; 1992.

[9] Dehaene S, Bossini S, Giraux P. The Mental Representation of Parity and Number Magnitude. Journal of Experimental Psychology: General. 1993;122(3):371–396. doi: 10.1037/0096-3445.122.3.371

[10] Nieder A, Diester I, Tudusciuc O. Temporal and Spatial Enumeration Processes in the Primate Parietal Cortex. Science. 2006;313(5792):1431–1435. doi: 10.1126/science.1130308 16960005

[11] Rugani R, Vallortigara G, Priftis K, Regolin L. Number-Space Mapping in the Newborn Chick Resembles Humans’ Mental Number Line. Science. 2015;347(6221):534–536. doi: 10.1126/science.aaa1379 25635096

[12] Gazes RP, Diamond RFL, Hope JM, Caillaud D, Stoinski TS, Hampton RR. Spatial Representation of Magnitude in Gorillas and Orangutans. Cognition. 2017;168:312–319. doi: 10.1016/j.cognition.2017.07.010 28772188

[13] Rugani R, Vallortigara G, Priftis K, Regolin L. Numerical Magnitude, Rather Than Individual Bias, Explains Spatial Numerical Association in Newborn Chicks. eLife. 2020;9:e54662. doi: 10.7554/eLife.54662 32584257 PMC7316507

[14] de Hevia MD, Veggiotti L, Streri A, Bonn CD. At Birth, Humans Associate “Few” with Left and “Many” with Right. Current Biology. 2017;27(24):3879–3884.e2. doi: 10.1016/j.cub.2017.11.024 29225024

[15] Di Giorgio E, Lunghi M, Rugani R, Regolin L, Dalla Barba B, Vallortigara G, et al. A Mental Number Line in Human Newborns. Developmental Science. 2019;22:e12801. doi: 10.1111/desc.12801 30676679

[16] Pica P, Lemer C, Izard V, Dehaene S. Exact and Approximate Arithmetic in an Amazonian Indigene Group. Science. 2004;306(5695):499–503. doi: 10.1126/science.1102085 15486303

[17] Zebian S. Linkages between Number Concepts, Spatial Thinking, and Directionality of Writing: The SNARC Effect and the Reverse SNARC Effect in English and Arabic Monoliterates, Biliterates, and Illiterate Arabic Speakers. Journal of Cognition and Culture. 2005;5(1–2):165–190. doi: 10.1163/1568537054068660

[18] Shaki S, Fischer MH, Göbel SM. Direction Counts: A Comparative Study of Spatially Directional Counting Biases in Cultures with Different Reading Directions. Journal of Experimental Child Psychology. 2012;112(2):275–281. doi: 10.1016/j.jecp.2011.12.005 22341408

[19] Shaki S, Fischer MH. Reading Space into Numbers—A Cross-Linguistic Comparison of the SNARC Effect. Cognition. 2008;108(2):590–599. doi: 10.1016/j.cognition.2008.04.001 18514179

[20] Fischer MH, Shaki S, Cruise A. It Takes Just One Word to Quash a SNARC. Experimental Psychology. 2009;56(5):361–366. doi: 10.1027/1618-3169.56.5.361 19447752

[21] Ito Y, Hatta T. Spatial Structure of Quantitative Representation of Numbers: Evidence from the SNARC Effect. Memory & Cognition. 2004;32(4):662–673. doi: 10.3758/BF03195857 15478760

[22] Nuerk HC, Wood G, Willmes K. The Universal SNARC Effect: The Association between Number Magnitude and Space Is Amodal. Experimental Psychology. 2005;52(3):187–194. doi: 10.1027/1618-3169.52.3.187 16076066

[23] Fischer MH. Spatial Representations in Number Processing: Evidence from a Pointing Task. Visual Cognition. 2003;10(4):493–508. doi: 10.1080/13506280244000186

[24] Shaki S, Petrusic WM. On the Mental Representation of Negative Numbers: Context-Dependent SNARC Effects with Comparative Judgments. Psychonomic Bulletin & Review. 2005;12(5):931–937. doi: 10.3758/BF03196788 16524013

[25] Patro K, Haman M. The Spatial–Numerical Congruity Effect in Preschoolers. Journal of Experimental Child Psychology. 2012;111(3):534–542. doi: 10.1016/j.jecp.2011.09.006 22153910

[26] Patro K, Shaki S. SNARC for Numerosities Is Modulated by Comparative Instruction (and Resembles Some Non-Numerical Effects). Cognitive Processing. 2016;17(2):127–137. doi: 10.1007/s10339-015-0745-2 26714804

[27] Gevers W, Reynvoet B, Fias W. The Mental Representation of Ordinal Sequences is Spatially Organised: Evidence from Days of the Week. Cortex. 2004;40(1):171–172. doi: 10.1016/S0010-9452(08)70938-9 15174454

[28] Guida A, Megreya AM, Lavielle-Guida M, Noël Y, Mathy F, van Dijck JP, et al. Spatialization in Working Memory is Related to Literacy and Reading Direction: Culture “Literarily” Directs Our Thoughts. Cognition. 2018;175:96–100. doi: 10.1016/j.cognition.2018.02.013 29486378

[29] Barth H, La Mont K, Lipton J, Dehaene S, Kanwisher N, Spelke E. Non-Symbolic Arithmetic in Adults and Young Children. Cognition. 2006;98(3):199–222. doi: 10.1016/j.cognition.2004.09.011 15876429

[30] Knops A, Viarouge A, Dehaene S. Dynamic Representations Underlying Symbolic and Nonsymbolic Calculation: Evidence from the Operational Momentum Effect. Attention, Perception, & Psychophysics. 2009;71(4):803–821. doi: 10.3758/APP.71.4.803 19429960

[31] Fischer MH. Cognition in the Bisection Task. Trends in Cognitive Sciences. 2001;5(11):460–462. doi: 10.1016/S1364-6613(00)01790-3 11684466

[32] Barrett AM, Kim M, Crucian GP, Heilman KM. Spatial Bias: Effects of Early Reading Direction on Korean Subjects. Neuropsychologia. 2002;40(7):1003–1012. doi: 10.1016/S0028-3932(01)00147-6 11900752

[33] Kazandjian S, Cavézian C, Zivotofsky AZ, Chokron S. Bisections in Two Languages: When Number Processing, Spatial Representation, and Habitual Reading Direction Interact. Neuropsychologia. 2010;48(14):4031–4037. doi: 10.1016/j.neuropsychologia.2010.10.020 20965204

[34] Fias W, Brysbaert M, Geypens F, d’Ydewalle G. The Importance of Magnitude Information in Numerical Processing: Evidence from the SNARC Effect. Mathematical Cognition. 1996;2(1):95–110. doi: 10.1080/135467996387552

[35] Schwarz W, Keus IM. Moving the Eyes along the Mental Number Line: Comparing SNARC Effects with Saccadic and Manual Responses. Perception & Psychophysics. 2004;66(4):651–664. doi: 10.3758/BF03194909 15311664

[36] Masson N, Andres M, Alsamour M, Bollen Z, Pesenti M. Spatial Biases in Mental Arithmetic Are Independent of Reading/Writing Habits: Evidence from French and Arabic Speakers. Cognition. 2020;200:104262. doi: 10.1016/j.cognition.2020.104262 32480066

[37] Nuerk HC, Iversen W, Willmes K. Notational Modulation of the SNARC and the MARC (Linguistic Markedness of Response Codes) Effect. Quarterly Journal of Experimental Psychology (Section A: Human Experimental Psychology). 2004;57(5):835–863. doi: 10.1080/02724980343000512 15204120

[38] Pansky A, Algom D. Comparative Judgment of Numerosity and Numerical Magnitude: Attention Preempts Automaticity. Journal of Experimental Psychology: Learning, Memory, and Cognition. 2002;28(2):259–274. 11911383 10.1037//0278-7393.28.2.259

[39] Mitchell T, Bull R, Cleland AA. Implicit Response-Irrelevant Number Information Triggers the SNARC Effect: Evidence Using a Neural Overlap Paradigm. Quarterly Journal of Experimental Psychology. 2012;65(10):1945–1961. doi: 10.1080/17470218.2012.673631 22524699

[40] Rusconi E, Kwan B, Giordano BL, Umilt C, Butterworth B. Spatial Representation of Pitch Height: The SMARC Effect. Cognition. 2006;99(2):113–129. doi: 10.1016/j.cognition.2005.01.004 15925355

[41] Shaki S, Petrusic WM, Leth-Steensen C. SNARC Effects with Numerical and Non-Numerical Symbolic Comparative Judgments: Instructional and Cultural Dependencies. Journal of Experimental Psychology: Human Perception and Performance. 2012;38(2):515–530. 22288694 10.1037/a0026729

[42] Dehaene S, Piazza M, Pinel P, Cohen L. Three Parietal Circuits for Number Processing. Cognitive Neuropsychology. 2003;20(3–6):487–506. doi: 10.1080/02643290244000239 20957581

[43] de Hevia MD, Girelli L, Macchi Cassia V. Minds without Language Represent Number through Space: Origins of the Mental Number Line. Frontiers in Psychology. 2012;3:article 466. doi: 10.3389/fpsyg.2012.00466 23118732 PMC3484654

[44] van Dijck JP, Fias W. A Working Memory Account for Spatial–Numerical Associations. Cognition. 2011;119(1):114–119. doi: 10.1016/j.cognition.2010.12.013 21262509

[45] Toomarian EY, Hubbard EM. On the Genesis of Spatial-Numerical Associations: Evolutionary and Cultural Factors Co-Construct the Mental Number Line. Neuroscience & Biobehavioral Reviews. 2018;90:184–199. doi: 10.1016/j.neubiorev.2018.04.010 29684402 PMC5993626

[46] Ginsburg V, Gevers W. Spatial Coding of Ordinal Information in Short- and Long-Term Memory. Frontiers in Human Neuroscience. 2015;9:8. doi: 10.3389/fnhum.2015.00008 25688199 PMC4311612

[47] Dehaene S. Varieties of Numerical Abilities. Cognition. 1992;44(1–2):1–42. 1511583 10.1016/0010-0277(92)90049-n

[48] Feigenson L, Dehaene S, Spelke E. Core Systems of Number. Trends in Cognitive Sciences. 2004;8(7):307–314. doi: 10.1016/j.tics.2004.05.002 15242690

[49] Ansari D. Does the Parietal Cortex Distinguish between “10,” “Ten,” and Ten Dots? Neuron. 2007;53(2):165–167. doi: 10.1016/j.neuron.2007.01.001 17224400

[50] Piazza M, Pinel P, LeBihan D, Dehaene S. A Magnitude Code Common to Numerosities and Number Symbols in Human Intraparietal Cortex. Neuron. 2007;53(2):293–305. doi: 10.1016/j.neuron.2006.11.022 17224409

[51] Fagard J, Dahmen R. The Effects of Reading-Writing Direction on the Asymmetry of Space Perception and Directional Tendencies: A Comparison between French and Tunisian Children. Laterality: Asymmetries of Brain, Behaviour, and Cognition. 2003;8(1):39–52. doi: 10.1080/713754473 15513214

[52] Shaki S, Fischer MH, Petrusic WM. Reading Habits for Both Words and Numbers Contribute to the SNARC Effect. Psychonomic Bulletin & Review. 2009;16(2):328–331. doi: 10.3758/PBR.16.2.328 19293102

[53] Göbel SM, Shaki S, Fischer MH. The Cultural Number Line: A Review of Cultural and Linguistic Influences on the Development of Number Processing. Journal of Cross-Cultural Psychology. 2011;42(4):543–556. doi: 10.1177/0022022111406251

[54] Pitt B, Ferrigno S, Cantlon JF, Casasanto D, Gibson E, Piantadosi ST. Spatial Concepts of Number, Size, and Time in an Indigenous Culture. Science Advances. 2021;7(33):eabg4141. doi: 10.1126/sciadv.abg4141 34380617 PMC8357228

[55] de Hevia MD, Spelke ES. Number-Space Mapping in Human Infants. Psychological Science. 2010;21(5):653–660. doi: 10.1177/0956797610366091 20483843 PMC3129621

[56] de Hevia MD, Izard V, Coubart A, Spelke ES, Streri A. Representations of Space, Time, and Number in Neonates. Proceedings of the National Academy of Sciences of the United States of America. 2014;111(13):4809–4813. doi: 10.1073/pnas.1323628111 24639511 PMC3977279

[57] Cantlon JF, Brannon EM. How Much Does Number Matter to a Monkey (*Macaca mulatta*)? Journal of Experimental Psychology: Animal Behavior Processes. 2007;33(1):32–41. 17227193 10.1037/0097-7403.33.1.32

[58] Zohar-Shai B, Tzelgov J, Karni A, Rubinsten O. It Does Exist! A Left-to-Right Spatial-Numerical Association of Response Codes (SNARC) Effect Among Native Hebrew Speakers. Journal of Experimental Psychology: Human Perception and Performance. 2017;43(4):719–728. 28182477 10.1037/xhp0000336

[59] Göbel SM, McCrink K, Fischer MH, Shaki S. Observation of Directional Storybook Reading Influences Young Children’s Counting Direction. Journal of Experimental Child Psychology. 2018;166:49–66. doi: 10.1016/j.jecp.2017.08.001 28865295 PMC5696009

[60] Feldman A, Oscar-Strom Y, Tzelgov J, Berger A. Spatial–Numerical Association of Response Code Effect as a Window to Mental Representation of Magnitude in Long-Term Memory among Hebrew-Speaking Children. Journal of Experimental Child Psychology. 2019;181:102–109. doi: 10.1016/j.jecp.2019.01.001 30735908

[61] Shaki S, Fischer MH. Removing Spatial Responses Reveals Spatial Concepts—Even in a Culture with Mixed Reading Habits. Frontiers in Human Neuroscience. 2014;8:966. doi: 10.3389/fnhum.2014.00966 25505403 PMC4244537

[62] Champely S. Pwr: Basic Functions for Power Analysis [R Package]; 2018.

[63] R Development Core Team. R: A Language and Environment for Statistical Computing; 2018.

[64] Gebuis T, Reynvoet B. Generating Nonsymbolic Number Stimuli. Behavior Research Methods. 2011;43(4):981–986. doi: 10.3758/s13428-011-0097-5 21512872

[65] Trick LM, Pylyshyn ZW. What Enumeration Studies Can Show Us about Spatial Attention: Evidence for Limited Capacity Preattentive Processing. Journal of Experimental Psychology: Human Perception and Performance. 1993;19(2):331–351. 8473843 10.1037//0096-1523.19.2.331

[66] Trick LM, Pylyshyn ZW. Why Are Small and Large Numbers Enumerated Differently? A Limited-Capacity Preattentive Stage in Vision. Psychological Review. 1994;101(1):80–102. doi: 10.1037/0033-295X.101.1.80 8121961

[67] Katzin N, Cohen ZZ, Henik A. If It Looks, Sounds, or Feels Like Subitizing, Is It Subitizing? A Modulated Definition of Subitizing. Psychonomic Bulletin & Review. 2019;26(3):790–797. doi: 10.3758/s13423-018-1556-0 30632105

[68] Leibovich-Raveh T, Lewis DJ, Al-Rubaiey Kadhim S, Ansari D. A New Method for Calculating Individual Subitizing Ranges. Journal of Numerical Cognition. 2018;4(2):429–447. doi: 10.5964/jnc.v4i2.74

[69] Maldonado Moscoso PA, Castaldi E, Burr DC, Arrighi R, Anobile G. Grouping Strategies in Number Estimation Extend the Subitizing Range. Scientific Reports. 2020;10(1):14979. doi: 10.1038/s41598-020-71871-5 32917941 PMC7486368

[70] Veale JF. Edinburgh Handedness Inventory—Short Form: A Revised Version Based on Confirmatory Factor Analysis. Laterality: Asymmetries of Brain, Behaviour, and Cognition. 2014;19(2):164–177. doi: 10.1080/1357650X.2013.783045 23659650

[71] Mathôt S, Schreij D, Theeuwes J. OpenSesame: An Open-Source, Graphical Experiment Builder for the Social Sciences. Behavior Research Methods. 2012;44(2):314–324. doi: 10.3758/s13428-011-0168-7 22083660 PMC3356517

[72] Kuznetsova A, Brockhoff PB, Christensen RHB. lmerTest Package: Tests in Linear Mixed Effects Models. Journal of Statistical Software. 2017;82(13):1–26. doi: 10.18637/jss.v082.i13

[73] Moyer RS, Landauer TK. Time Required for Judgements of Numerical Inequality. Nature. 1967;215(5109):1519–1520. doi: 10.1038/2151519a0 6052760

[74] Kliegl R, Wei P, Dambacher M, Yan M, Zhou X. Experimental Effects and Individual Differences in Linear Mixed Models: Estimating the Relationship between Spatial, Object, and Attraction Effects in Visual Attention. Frontiers in Psychology. 2011;1:article 238. doi: 10.3389/fpsyg.2010.00238 21833292 PMC3153842

[75] Fox J, Weisberg S. An R Companion to Applied Regression. 3rd ed. Thousand Oaks, CA: Sage Publications; 2019.

[76] de Bot K, Clyne M. A 16-Year Longitudinal Study of Language Attrition in Dutch Immigrants in Australia. Journal of Multilingual and Multicultural Development. 1994;15(1):17–28. doi: 10.1080/01434632.1994.9994554

[77] Beganović J. First Language Attrition and Syntactic Subjects: A Study of Serbian, Croatian, and Bosnian Intermediate and Advanced Speakers of Dutch. University of Edinburgh. Edinburgh, UK; 2006.

[78] Wood G, Willmes K, Nuerk HC, Fischer MH. On the Cognitive Link between Space and Number: A Meta-Analysis of the SNARC Effect. Psychology Science Quarterly. 2008;50(4):489–525.

[79] Cipora K, Nuerk HC. Is the SNARC Effect Related to the Level of Mathematics? No Systematic Relationship Observed Despite More Power, More Repetitions, and More Direct Assessment of Arithmetic Skill. Quarterly Journal of Experimental Psychology. 2013;66(10):1974–1991. doi: 10.1080/17470218.2013.772215 23473520

[80] Ansari D. Effects of Development and Enculturation on Number Representation in the Brain. Nature Reviews Neuroscience. 2008;9(4):278–291. doi: 10.1038/nrn2334 18334999

[81] Zebian S, Ansari D. Differences between Literates and Illiterates on Symbolic but Not Nonsymbolic Numerical Magnitude Processing. Psychonomic Bulletin & Review. 2012;19(1):93–100. doi: 10.3758/s13423-011-0175-9 22033982

[82] Piazza M, Izard V, Pinel P, Le Bihan D, Dehaene S. Tuning Curves for Approximate Numerosity in the Human Intraparietal Sulcus. Neuron. 2004;44(3):547–555. doi: 10.1016/j.neuron.2004.10.014 15504333

[83] Revkin SK, Piazza M, Izard V, Cohen L, Dehaene S. Does Subitizing Reflect Numerical Estimation? Psychological Science. 2008;19(6):607–614. doi: 10.1111/j.1467-9280.2008.02130.x 18578852

[84] Zhou X, Shen C, Li L, Li D, Cui J. Mental Numerosity Line in the Human’s Approximate Number System. Experimental Psychology. 2016;6(3):169–179. doi: 10.1027/1618-3169/a000324 27404985

[85] Nemeh F, Humberstone J, Yates MJ, Reeve RA. Non-Symbolic Magnitudes Are Represented Spatially: Evidence from a Non-Symbolic SNARC Task. PLoS ONE. 2018;13(8):e0203019. doi: 10.1371/journal.pone.0203019 30161171 PMC6116986

[86] Cleland AA, Corsico K, White K, Bull R. Non-Symbolic Numerosities Do Not Automatically Activate Spatial–Numerical Associations: Evidence from the SNARC Effect. Quarterly Journal of Experimental Psychology. 2020;73(2):295–308. doi: 10.1177/1747021819875021 31432745

[87] Dehaene S, Dupoux E, Mehler J. Is Numerical Comparison Digital? Analogical and Symbolic Effects in Two-Digit Number Comparison. Journal of Experimental Psychology: Human Perception and Performance. 1990;16(3):626–641. 2144576 10.1037//0096-1523.16.3.626

[88] Tselgov J, Meyer J, Henik A. Automatic and Intentional Processing of Numerical Information. Journal of Experimental Psychology: Learning, Memory, and Cognition. 1992;18(1):166–179.

[89] Gevers W, Verguts T, Reynvoet B, Caessens B, Fias W. Numbers and Space: A Computational Model of the SNARC Effect. Journal of Experimental Psychology: Human Perception and Performance. 2006;32(1):32–44. 16478324 10.1037/0096-1523.32.1.32

[90] Cipora K, Wood G. Finding the SNARC Instead of Hunting It: A 20*20 Monte Carlo Investigation. Frontiers in Psychology. 2017;8:1194. doi: 10.3389/fpsyg.2017.01194 28769840 PMC5513957

[91] Grosjean F, Li P. The Psycholinguistics of Bilingualism. Malden, MA: Blackwell Publishing; 2013.

[92] Cipora K, Soltanlou M, Reips UD, Nuerk HC. The SNARC and MARC Effects Measured Online: Large-Scale Assessment Methods in Flexible Cognitive Effects. Behavior Research Methods. 2019;51(4):1676–1692. doi: 10.3758/s13428-019-01213-5 30805864

[93] Fischer MH, Mills RA, Shaki S. How to Cook a SNARC: Number Placement in Text Rapidly Changes Spatial–Numerical Associations. Brain and Cognition. 2010;72(3):333–336. doi: 10.1016/j.bandc.2009.10.010 19917517

