# Supplementary material for: Cultural factors weaken but do not reverse left-to-right spatial biases in numerosity processing: Data from Arabic and English monoliterates and Arabic-English biliterates
Source: PLoS One. 2021 Dec 16;16(12):e0261146. doi: 10.1371/journal.pone.0261146 (PMC8675726; doi:10.1371/journal.pone.0261146)
Supplement: S1 Table — (PDF) [file pone.0261146.s001.pdf]

Supporting information

S1 Table. Fixed effects in Model 1A (intercept represents Group = EM, Size = small, Condition = *smaller*).

**Predictor**	β	SE	t	p	
(Intercept)	28.483	13.295	2.142	.033	*
Group: AM	-39.324	23.615	-1.665	.096	†
Group: AEBUS	-8.364	18.266	-0.458	.647	
Group: AEBJO	-28.868	20.797	-1.388	.165	
Size: cross-range	-0.825	17.864	-0.046	.963	
Size: large	-61.849	17.895	-3.456	$<$.001	***
Condition: *larger*	-45.925	17.465	-2.629	.009	**
Group: AM × Size: cross-range	17.468	31.659	0.552	.581	
Group: AEBUS × Size: cross-range	17.164	24.472	0.701	.483	
Group: AEBJO × Size: cross-range	43.483	27.881	1.560	.119	
Group: AM × Size: large	23.238	31.676	0.734	.463	
Group: AEBUS × Size: large	0.041	24.494	0.002	.999	
Group: AEBJO × Size: large	26.826	27.901	0.961	.336	
Group: AM × Condition: *larger*	57.113	31.659	1.804	.071	†
Group: AEBUS × Condition: *larger*	-1.107	24.472	-0.045	.964	
Group: AEBJO × Condition: *larger*	15.319	27.881	0.549	.583	
Size: cross-range × Condition: *larger*	6.216	24.700	0.252	.801	
Size: large × Condition: *larger*	113.424	24.722	4.588	$<$.001	***
Group: AM × Size: cross-range × Condition: *larger*	-36.706	44.772	-0.820	.412	
Group: AEBUS × Size: cross-range × Condition: *larger*	-22.658	34.609	-0.655	.513	
Group: AEBJO × Size: cross-range × Condition: *larger*	-49.403	39.430	-1.253	.210	
Group: AM × Size: large × Condition: *larger*	-47.638	44.784	-1.064	.288	
Group: AEBUS × Size: large × Condition: *larger*	-6.605	34.610	-0.191	.849	
Group: AEBJO × Size: large × Condition: *larger*	-48.288	39.444	-1.224	.221	

Note. Significance codes: † $p < .1$; * $p < .05$; ** $p < .01$; *** $p < .001$.
