# Supplementary material for: Cultural factors weaken but do not reverse left-to-right spatial biases in numerosity processing: Data from Arabic and English monoliterates and Arabic-English biliterates
Source: PLoS One. 2021 Dec 16;16(12):e0261146. doi: 10.1371/journal.pone.0261146 (PMC8675726; doi:10.1371/journal.pone.0261146)
Supplement: S2 Table — (PDF) [file pone.0261146.s002.pdf]

Supporting information

S2 Table. Fixed effects in Model 1B (intercept represents Group = AM, Size = small, Condition = *smaller*).

**Predictor**	β	SE	t	p	
(Intercept)	-10.841	20.331	-0.533	.594	
Group: EM	39.324	23.754	1.655	.098	†
Group: AEBUS	30.947	23.630	1.310	.191	
Group: AEBJO	10.456	25.660	0.407	.684	
Size: cross-range	16.643	27.271	0.610	.542	
Size: large	-38.611	27.271	-1.416	.158	
Condition: *larger*	11.188	26.497	0.422	.673	
Group: EM × Size: cross-range	-17.468	31.768	-0.550	.583	
Group: AEBUS × Size: cross-range	-0.292	31.590	-0.009	.993	
Group: AEBJO × Size: cross-range	26.015	34.317	0.758	.449	
Group: EM × Size: large	-23.213	31.786	-0.730	.465	
Group: AEBUS × Size: large	-23.185	31.590	-0.734	.463	
Group: AEBJO × Size: large	3.588	34.317	0.105	.917	
Group: EM × Condition: *larger*	-57.113	31.768	-1.798	.072	†
Group: AEBUS × Condition: *larger*	-58.207	31.590	-1.843	.066	†
Group: AEBJO × Condition: *larger*	-41.794	34.317	-1.218	.223	
Size: cross-range × Condition: *larger*	-30.489	37.472	-0.814	.416	
Size: large × Condition: *larger*	65.786	37.472	1.756	.079	†
Group: EM × Size: cross-range × Condition: *larger*	36.706	44.927	0.817	.414	
Group: AEBUS × Size: cross-range × Condition: *larger*	14.049	44.676	0.314	.753	
Group: AEBJO × Size: cross-range × Condition: *larger*	-12.697	48.532	-0.262	.794	
Group: EM × Size: large × Condition: *larger*	47.613	44.939	1.059	.290	
Group: AEBUS × Size: large × Condition: *larger*	41.021	44.664	0.918	.359	
Group: AEBJO × Size: large × Condition: *larger*	-0.650	48.532	-0.013	.989	

Note. Significance code: † $p < .1$.
