# Supplementary material for: Cultural factors weaken but do not reverse left-to-right spatial biases in numerosity processing: Data from Arabic and English monoliterates and Arabic-English biliterates
Source: PLoS One. 2021 Dec 16;16(12):e0261146. doi: 10.1371/journal.pone.0261146 (PMC8675726; doi:10.1371/journal.pone.0261146)
Supplement: S3 Table — (PDF) [file pone.0261146.s003.pdf]

Supporting information

S3 Table. Fixed effects in Model 1C (intercept represents Group = AEBUS, Size = small, Condition = *smaller*).

**Predictor**	β	SE	t	p	
(Intercept)	20.106	13.664	1.472	.144	
Group: EM	8.377	18.374	0.456	.649	
Group: AM	-30.947	23.630	-1.310	.191	
Group: AEBJO	-20.491	20.779	-0.986	.324	
Size: cross-range	16.351	18.372	0.890	.376	
Size: large	-61.796	18.372	-3.364	.001	**
Condition: *larger*	-47.019	17.201	-2.733	.006	**
Group: EM × Size: cross-range	-17.176	24.557	-0.699	.484	
Group: AM × Size: cross-range	0.292	31.590	0.009	.993	
Group: AEBJO × Size: cross-range	26.307	27.775	0.947	.344	
Group: EM × Size: large	-0.028	24.579	-0.001	.999	
Group: AM × Size: large	23.185	31.590	0.734	.463	
Group: AEBJO × Size: large	26.773	27.775	0.964	.335	
Group: EM × Condition: *larger*	1.095	24.557	0.045	.964	
Group: AM × Condition: *larger*	58.207	31.590	1.843	.066	†
Group: AEBJO × Condition: *larger*	16.414	27.775	0.591	.555	
Size: cross-range × Condition: *larger*	-16.440	24.326	-0.676	.499	
Size: large × Condition: *larger*	106.807	24.305	4.394	$<$.001	***
Group: EM × Size: cross-range × Condition: *larger*	22.657	34.729	0.652	.514	
Group: AM × Size: cross-range × Condition: *larger*	-14.049	44.675	-0.314	.753	
Group: AEBJO × Size: cross-range × Condition: *larger*	-26.746	39.280	-0.681	.496	
Group: EM × Size: large × Condition: *larger*	6.592	34.730	0.190	.849	
Group: AM × Size: large × Condition: *larger*	-41.021	44.664	-0.918	.358	
Group: AEBJO × Size: large × Condition: *larger*	-41.671	39.268	-1.061	.289	

Note. Significance codes: † $p < .1$; ** $p < .01$; *** $p < .001$.
