# Supplementary material for: Cultural factors weaken but do not reverse left-to-right spatial biases in numerosity processing: Data from Arabic and English monoliterates and Arabic-English biliterates
Source: PLoS One. 2021 Dec 16;16(12):e0261146. doi: 10.1371/journal.pone.0261146 (PMC8675726; doi:10.1371/journal.pone.0261146)
Supplement: S4 Table — (PDF) [file pone.0261146.s004.pdf]

## Supporting information

**S4 Table. Fixed effects in Model 1D (intercept represents Group = AEBJO, Size = small, Condition = *smaller*).**

| **Predictor** | $\beta$ | $SE$ | $t$ | $p$ | |
|---|---|---|---|---|---|
| (Intercept) | -0.385 | 16.933 | -0.023 | .982 | |
| Group: EM | 28.868 | 20.919 | 1.380 | .168 | |
| Group: AM | -10.456 | 25.660 | -0.407 | .684 | |
| Group: AEBUS | 20.491 | 20.779 | 0.986 | .324 | |
| Size: cross-range | 42.658 | 22.743 | 1.876 | .062 | † |
| Size: large | -35.023 | 22.743 | -1.540 | .125 | |
| Condition: *larger* | -30.606 | 21.808 | -1.403 | .161 | |
| Group: EM × Size: cross-range | -43.483 | 27.978 | -1.554 | .120 | |
| Group: AM × Size: cross-range | -26.015 | 34.317 | -0.758 | .449 | |
| Group: AEBUS × Size: cross-range | -26.307 | 27.776 | -0.947 | .344 | |
| Group: EM × Size: large | -26.800 | 27.997 | -0.957 | .339 | |
| Group: AM × Size: large | -3.588 | 34.317 | -0.105 | .917 | |
| Group: AEBUS × Size: large | -26.773 | 27.776 | -0.964 | .335 | |
| Group: EM × Condition: *larger* | -15.319 | 27.978 | -0.548 | .584 | |
| Group: AM × Condition: *larger* | 41.794 | 34.317 | 1.218 | .223 | |
| Group: AEBUS × Condition: *larger* | -16.414 | 27.776 | -0.591 | .555 | |
| Size: cross-range × Condition: *larger* | -43.186 | 30.841 | -1.400 | .162 | |
| Size: large × Condition: *larger* | 65.136 | 30.841 | 2.112 | .035 | * |
| Group: EM × Size: cross-range × Condition: *larger* | 49.403 | 39.566 | 1.249 | .212 | |
| Group: AM × Size: cross-range × Condition: *larger* | 12.697 | 48.532 | 0.262 | .794 | |
| Group: AEBUS × Size: cross-range × Condition: *larger* | 26.746 | 39.280 | 0.681 | .496 | |
| Group: EM × Size: large × Condition: *larger* | 48.263 | 39.580 | 1.219 | .223 | |
| Group: AM × Size: large × Condition: *larger* | 0.650 | 48.532 | 0.013 | .989 | |
| Group: AEBUS × Size: large × Condition: *larger* | 41.671 | 39.268 | 1.061 | .289 | |

Note. Significance codes: † $p < .1$; * $p < .05$.
