# Supplementary material for: Cultural factors weaken but do not reverse left-to-right spatial biases in numerosity processing: Data from Arabic and English monoliterates and Arabic-English biliterates
Source: PLoS One. 2021 Dec 16;16(12):e0261146. doi: 10.1371/journal.pone.0261146 (PMC8675726; doi:10.1371/journal.pone.0261146)
Supplement: S5 Table — (PDF) [file pone.0261146.s005.pdf]

Supporting information

S5 Table. Fixed effects in Model 3 (intercept represents CountLang = English, Size = small, Condition = *smaller*).

**Predictor**	β	SE	t	p	
(Intercept)	17.732	14.658	1.210	.229	
CountLang: Arabic	-22.908	22.281	-1.028	.304	
CountLang: English+Arabic	33.958	38.377	0.885	.377	
Size: cross-range	6.676	19.052	0.350	.727	
Size: large	-67.689	19.052	-3.553	<.001	***
Condition: *larger*	-32.337	18.883	-1.712	.087	†
CountLang: Arabic × Size: cross-range	50.204	28.943	1.735	.083	†
CountLang: English+Arabic × Size: cross-range	12.570	49.873	0.252	.801	
CountLang: Arabic × Size: large	63.587	28.943	2.197	.028	*
CountLang: English+Arabic × Size: large	-86.676	49.873	-1.738	.082	†
CountLang: Arabic × Condition: *larger*	-5.457	28.943	-0.189	.850	
CountLang: English+Arabic × Condition: *larger*	-72.743	49.873	-1.459	.145	
Size: cross-range × Condition: *larger*	-8.538	26.678	-0.320	.749	
Size: large × Condition: *larger*	106.719	26.678	4.000	<.001	***
CountLang: Arabic × Size: cross-range × Condition: *larger*	-53.885	40.914	-1.317	.188	
CountLang: English+Arabic × Size: cross-range × Condition: *larger*	20.342	70.907	0.287	.774	
CountLang: Arabic × Size: large × Condition: *larger*	-75.307	40.914	-1.841	.066	†
CountLang: English+Arabic × Size: large × Condition: *larger*	124.654	70.520	1.768	.077	†

Note. Significance codes: † $p < .1$; * $p < .05$; *** $p < .001$.
